# Rectovaginal Fistula in a 10-Year-Old With Hirschsprung Disease: A Case Report

**DOI:** 10.7759/cureus.57316

**Published:** 2024-03-31

**Authors:** Jomaries O Gomez Rosado, Courteney Castellano, Jossias Genao Cruz, Jonas Esgusquiza

**Affiliations:** 1 College of Medicine, Nova Southeastern University Dr. Kiran C. Patel College of Osteopathic Medicine, Fort Lauderdale, USA; 2 Department of Pediatrics, Broward Health Medical Center, Fort Lauderdale, USA

**Keywords:** hirschsprung disease (hd), colonic ulcers, incontinence, colonoscopy, rectovaginal fistula

## Abstract

Hirschsprung disease is an uncommon medical condition caused by the lack of migration of ganglion cells to the rectum during embryonic development, affecting the peristaltic movements of the intestine. It is a chronic medical condition responsible for chronic constipation and intestinal obstruction. We present the case of a 10-year-old female with a history of Hirschsprung disease and colectomy admitted to a pediatric hospital for the management of multiple colonic ulcers and severe anemia who subsequently developed a rectovaginal fistula. This patient's admission was complicated by perianal and vaginal excoriations, a paralytic ileus, and fecal incontinence. This case report is unique due to the development of a rare pediatric complication of Hirschsprung disease.

## Introduction

Hirschsprung disease (HD) is a congenital disorder characterized by the absence of ganglion cells on the terminal rectum with variable extension through the intestines [1]. Hirschsprung disease is the main cause of functional intestinal obstruction, with an incidence of 1/500 live births and a variable prevalence of 1 to 1.63 per 10,000 births [1,2].

A rectovaginal fistula is a rare abnormal epithelial connection between the anterior wall of the rectum and the posterior wall of the vagina [3]. These fistulas can be caused by inflammatory bowel disease, trauma, or iatrogenic injury [3]. Rectovaginal fistulas are rare complications of Hirschsprung disease and are highly uncommon in children. Only a few cases have documented the development of fistulas in patients with Hirschsprung disease [4]. We present a case of a 10-year-old female with a previous medical history of Hirschsprung disease who presented with anemia and stool coming out of her vagina.

## Case presentation

A 10-year-old female with a past medical history of Hirschsprung disease and failure to thrive on gastrostomy tube feeds was admitted to our general pediatric unit at an urban city hospital with anemia due to multiple colonic ulcers. The patient was born at 36 weeks of gestation, secondary to maternal hypertension. Her birth history was complicated by respiratory distress at birth requiring positive pressure ventilation; however, she was never intubated. After birth, the patient had persistent bilious emesis, for which she received an exploratory laparotomy, revealing multiple meconium plugs. Her workup included a rectal biopsy, which was positive for Hirschsprung disease. Her Hirschsprung disease affected the entire colon, extending to the terminal ileum. Therefore, she underwent a total colon resection at one month of age, along with the placement of an ileostomy. Feedings were gradually advanced with success. By three years of age, the patient underwent a colostomy, an ileostomy takedown, and an endorectal pull-through was performed.

The patient regularly followed up with pediatric gastroenterology and nutrition. Recent visits were remarkable for severe anemia, leading to the scheduling of a colonoscopy and endoscopy for further evaluation. In an outpatient clinic the day prior to admission, the patient underwent an endoscopy and colonoscopy under general anesthesia to assess for any gastrointestinal tract bleeding as a cause for her anemia. The endoscopy revealed normal anatomy with no signs or sources of bleeding. Her colonoscopy showed ulcers and inflammation at 20 cm distal to the small bowel with rectal anastomosis. The surgical pathology report indicated that the small bowel biopsy at 20 cm showed small bowel-type mucosa with a focally denuded surface and no significant pathological changes. Additionally, no histopathology consistent with inflammatory bowel disease was noted throughout the report.

After the procedure, the patient was admitted to the pediatric inpatient unit for observation, anemia management, and intravenous antibiotic treatment. Upon admission, the patient was severely anemic, according to the laboratory findings listed in Table 1. The patient received a one-unit red blood cell transfusion. Rectal irrigations were discontinued to facilitate the healing of the colonic ulcers, and mesalamine rectal enemas were administered twice a day. She then received another two units of red blood cells; hemoglobin remained stable at 10.9 g/dL (normal: 11.5-14.5 g/dL) for the remainder of the stay.

**Table 1:** Laboratory results of the patient upon admission to the hospital MCV: mean corpuscular volume

Blood analysis on admission	Patient’s results	Normal range
Hemoglobin	6.2 g/dL	11.5-14.5 g/dL
Hematocrit	24.4%	35- 42%
MCV	65.1 fL	77-95 fL
Platelets	489 x 103 mL	250-550 x 103 mL

Post-procedure care was complicated by a paralytic ileus, which was managed with supportive care. This included bowel decompression through venting the gastrostomy tube, administering maintenance fluids, and implementing bowel rest by keeping the patient nothing by mouth (NPO). On the third day post-colonoscopy, the patient's mother noticed stool discharge from the vaginal canal. On the fourth day post-colonoscopy, the patient experienced continuous stool vaginal discharge, leading to perianal and vaginal excoriations managed with mupirocin. No stool was observed in the urine or the urethra. A voiding cystourethrogram (VCUG) was ordered to rule out a colonic-vesical fistula. The VCUG showed normal anatomy, normal voiding dynamics, and no evidence of contrast extravasation into the colon or surrounding structures.

On the seventh day following the colonoscopy procedure, the patient resumed her regular diet without any episodes of emesis or abdominal pain. She tolerated gentle rectal irrigations and experienced normal bowel movements. However, these bowel movements were still associated with vaginal stool discharge. The vaginal and perianal excoriations showed improvement from the initial presentation. Throughout her stay, mupirocin was applied as necessary to alleviate the vaginal and perianal irritation. The patient's parents scheduled follow-up appointments with both her surgeon and gastroenterologist for one week after discharge.

## Discussion

Hirschsprung disease is a congenital disorder of the gut defined by the absence of ganglion cells at Meissner's plexus of the submucosa and Auerbach's plexus of the muscularis in the terminal rectum [1]. The degree to which the absence of these ganglion cells extends varies between individuals. Failure of the migration of these ganglion cells results in the portion of the aganglionic segment of the colon failing to relax, resulting in a functional obstruction [5]. Hirschsprung disease presents mainly in the neonatal period, with 65% of the cases being diagnosed before the age of one month [6]. The diagnosis of Hirschsprung disease in the neonatal period involves recognizing features of intestinal obstruction, such as the failure to pass meconium within the first 48 hours of life, abdominal distension, vomiting, and neonatal enterocolitis [2].

Surgical correction is the primary approach for addressing Hirschsprung disease. A pull-through procedure involves surgically removing the segment of the bowel affected by Hirschsprung disease, connecting the healthy portion of the intestine to the anus, and releasing the tonic contraction of the internal anal sphincter [2]. This allows for the passage of stool through the newly created pathway. In cases of short-segment Hirschsprung disease, a pull-through procedure is often sufficient [2]. The timing for this procedure varies, typically occurring between four to six months following colostomy placement [2]. Various types of pull-through procedures exist, with Swenson's technique being a traditional option involving proctectomy, pulling the healthy ganglionated colon through, and anastomosing it to the anus [2]. In this case, the patient had Hirschsprung disease extending into the ileum, ultimately requiring a total colectomy, setting the stage for subsequent complications.

Fistulas are abnormal connections between two organs or tissues; they form when the integrity of the walls is compromised [7]. Rectovaginal fistulas can develop as a complication of chronic disease, inflammation, trauma, obstruction, and many other processes [7]. The development of fistulas in Hirschsprung has been associated with invasive procedures such as the Hirschsprung redo pull-through procedure, in which only 9.7% of patients with this disease develop fistulas after [4]. However, this was not the case with our patient.

In our case, the rectovaginal fistula was acquired after a colonoscopy. We postulate that the fistula occurred due to chronic inflammation associated with Hirschsprung disease, further exacerbated by the multiple anastomotic colonic ulcers and the post-colonoscopy paralytic ileus. Chronic inflammation can lead to erosion of both the colon and vaginal wall, resulting in the establishment of such abnormal connections [7]. 

In pediatrics, rectovaginal fistulas are rare. We estimate the incidence to be around 1% of reported fistulas [8]. A specific number is hard to provide given that incidence reports are associated with specific underlying causes and patient population [8-10]. 

The clinical presentation of a rectovaginal fistula includes signs of the passage of stool or gas from the rectum to the vagina; it can be associated with foul-smelling vaginal discharge, irritation, and swelling of the vaginal canal. These symptoms worsen with the passage of diarrhea [7]. Initially, the patient presented with some vaginal stool discharge, which became continuous as the patient developed loose stool. The continued stool vaginal discharge caused our patient to develop vaginal excoriations. Therefore, the diagnosis of a rectovaginal fistula was possible based on the clinical presentation and physical exam findings. 

Magnetic resonance imaging (MRI) is the best imaging modality to visualize this type of pathology [11]. The pediatric team recommended an MRI of the pelvis to further evaluate the rectovaginal fistula. However, given that the patient would need sedation with general anesthesia, we decided to postpone the study. The patient still had the symptomatology of a paralytic ileus, such as abdominal pain and distention. The use of general anesthesia promotes the development or worsening of an ileus [12]. It was a clinical decision to hold the study until the acute abdominal pain and distention fully resolved. The patient agreed to follow up with outpatient surgery and gastroenterology for further management.

Rectovaginal fistulas constitute only a small percentage of all fistulas; they account for less than 5% of all perianal fistulas [13]. As previously discussed, although rare, inflammatory bowel diseases, trauma, and obstruction are possible causes [3]. Regardless of the etiology, rectovaginal fistulas can significantly impact a patient's quality of life. Prompt recognition and management can help a patient cope with emotional distress and social impact. 

## Conclusions

Hirschsprung disease is an uncommon condition that is associated with various complications. Our case report highlights an unexpected complication of Hirschsprung, the development of a rectovaginal fistula. Rectovaginal fistulas are rare but treatable findings whose identification and management should be recognized in patients with Hirschsprung disease and prior surgical interventions.
